# Selective Eradication of *Staphylococcus aureus* by the Designer Genetically Programmed Yeast Biocontrol Agent

**DOI:** 10.3390/antibiotics9090527

**Published:** 2020-08-19

**Authors:** Sofiya O. Pipiya, Yuliana A. Mokrushina, Alexander G. Gabibov, Ivan V. Smirnov, Stanislav S. Terekhov

**Affiliations:** 1 Shemyakin-Ovchinnikov Institute of Bioorganic Chemistry, Russian Academy of Sciences, Moscow 117997, Russia; sonya-pipiya@yandex.ru (S.O.P.); yuliana256@mail.ru (Y.A.M.); 2 Department of Chemistry, Lomonosov Moscow State University, Moscow 119991, Russia; 3 Department of Life Sciences, Higher School of Economics, Moscow 101000, Russia

**Keywords:** *Staphylococcus aureus* eradication, yeast biocontrol agent, recombinant lysostaphin, genetically programmed probiotics, targeted lysis, constitutive heterologous production in *Pichia pastoris*

## Abstract

*Staphylococcus aureus* is a common human pathogen that is particularly often associated with antibiotic resistance. The eradication of this ubiquitous infectious agent from its ecological niches and contaminated surfaces is especially complicated by excessive biofilm formation and persisting cells, which evade the antibacterial activity of conventional antibiotics. Here, we present an alternative view of the problem of specific *S. aureus* eradication. The constitutive heterologous production of highly specific bacteriolytic protease lysostaphin in yeast *Pichia pastoris* provides an efficient biocontrol agent, specifically killing *S. aureus* in coculture. A yeast-based anti-*S. aureus* probiotic was efficient in a high range of temperatures and target-to-effector ratios, indicating its robustness and versatility in eliminating *S. aureus* cells. The efficient eradication of *S. aureus* by live lysostaphin-producing *P. pastoris* was achieved at high scales, providing a simple, biocompatible and cost-effective strategy for *S. aureus* lysis in bioproduction and surface decontamination. Future biomedical applications based on designer yeast biocontrol agents require evaluation in in vivo models. However, we believe that this strategy is very promising since it provides highly safe, efficient and selective genetically programmed probiotics and targeted biocontrol agents.

## 1. Introduction

Antibiotic resistance (AR) is a persistent threat to global healthcare, resulting in up to 11 million deaths annually [1,2]. *Staphylococcus aureus* is one of the most common pathogens, accounting for nearly 120,000 severe cases of bloodstream infections and 20,000 deaths per year in the United States alone [3]. *S. aureus* is a highly specialized human pathogen, notorious for an extensive number of methicillin-resistant (MRSA) strains resistant to the entire β-lactam antibiotic class [4]. Moreover, *S. aureus* strains have adopted a variety of virulence factors to efficiently colonize and counteract host immunity [5]. The exceptionally flexible *S. aureus* adaptation to antibiotics arises not only from the horizontal transfer of mobile genetic elements. It is also enhanced by increased mutation rates of *S. aureus* strains. The estimated mutation rate for the *S. aureus* isolate collection is 3.3 × 10^−6^ per site per year [6]. This rate is about 1000 times faster than the canonical substitution rate estimate for *E. coli* [7]. A high mutation rate of *S. aureus* isolates relates to their low doubling time in the wild [8]. This mutability provides the increased evolutionary space for *S. aureus* that it implements for its rapid adaptation. Hence, the eradication of *S. aureus* is an incredibly difficult task because of its plasticity, ubiquity, propensity to AR and simplicity of transmission [5]. Extensive biofilm formation and persisters in turn worsen the situation dramatically, making *S. aureus* non-sensitive to antibiotic drugs [9].

Bacteriolytic enzymes, disrupting cell integrity, represent a promising alternative to conventional antibiotics. The most promising example of the former is lysostaphin, which not only kills planktonic *S. aureus* but also disrupts *S. aureus* biofilms [10]. Lysostaphin is a specific zinc-containing metalloprotease secreted by strains of *S. simulans* biovar *staphylolyticus* [11]. Lysostaphin cleaves the pentaglycine crossbridges present in peptidoglycan (PG), the essential component of the bacterial cell wall, leading to rapid cell lysis. The recognition of the pentaglycine crossbridge and the peptide stem is shared by two independent SH3b domains, allowing protein clustering on the PG [12]. This unusual mechanism underpins the potent activity of lysostaphin, and its capacity to punch holes in the cell walls so as to cause rapid cell lysis.

Lysostaphin displays bacteriolytic activity against numerous *Staphylococcus* species [13,14]. It is highly active against virtually any natural *S. aureus* strain, including clinically relevant MRSA and vancomycin intermediately susceptible *S. aureus* (VISA) [15,16]. This makes lysostaphin a promising agent in the experimental therapy of *S. aureus* infections overperforming conventional vancomycin therapy in different animal models [17,18,19], thus providing an essential therapeutic agent against VISA and vancomycin-resistant *S. aureus* (VRSA) [20].

A wide palette of applications illustrates the versatility and efficiency of lysostaphin in the eradication of *S. aureus* from different environments. Hydrogel-based materials eliminating implant *S. aureus* infections represent an example of an advanced biomaterial facilitating healing [21]. The heterologous production of lysostaphin was applied to protect transgenic animals from mastitis, eradicating *S. aureus* from milk and mammary tissue [22]. Encapsulated mammalian cells with a synthetic gene circuit to sense and respond to MRSA infection could provide potential prophylactic, diagnostic or therapeutic options for treating medical implant-associated infections [23]. Deimmunized lysostaphin variants are valuable in this case as they expand the boundaries of lysostaphin systemic administration and therapy [24].

Here we describe an alternative paradigm of specific *S. aureus* eradication. We consider applying the high viability, robustness, and safety of yeasts to create a genetically programmed biocontrol agent, killing *S. aureus* cells and destroying its biofilms. The heterologous inducible production of recombinant lysostaphin in yeast *Pichia pastoris* was previously achieved via the codon optimization of its natural sequence [25]. Alternatively, we focused on the constitutive production of lysostaphin in yeast cells to obtain live cell cultures, eradicating *S. aureus*. *P. pastoris* produced up to 30 mg/L/day of recombinant lysostaphin in shaking flask conditions. This culture medium was highly active against *S. aureus*, leading to growth inhibition and bactericidal effects at 1.6 × 10^4^- and 4 × 10^3^-fold dilutions, respectively. Moreover, 10^7^ CFU/mL of *P. pastoris* eradicated up to 10^10^ CFU/mL *S. aureus* after 12 h in coculture. Recombinant lysostaphin-producing (rLys) *P. pastoris* was active against *S. vitulinus* and *S. xylosus*, which have similar PG crossbridge sequences, correlating with the lysostaphin activity spectrum. The anti-*S. aureus* activity of live rLys *P. pastoris* culture was prominent at the 20–33 °C temperature range, with some decrease at 37 °C that we associate with the elevated growth temperature, non-optimal for *P. pastoris*. The efficient eradication of *S. aureus* via engineered *P. pastoris* in coculture was achieved in a broad range of target (*S. aureus*):effector (rLys *P. pastoris*) ratios, both at high and low *S. aureus* cell densities. These results illustrate the considerable potential of live lysostaphin-producing cultures in *S. aureus* elimination, creating a background for their further applications in vivo. Yeast-based bacteriolytic live cultures represent a particularly promising approach to the creation of highly safe, efficient and selective genetically programmed probiotics and targeted designer biocontrol agents.

## 2. Results

### 2.1. Constitutive Production of Recombinant Lysostaphin in P. pastoris

**Figure 1:**
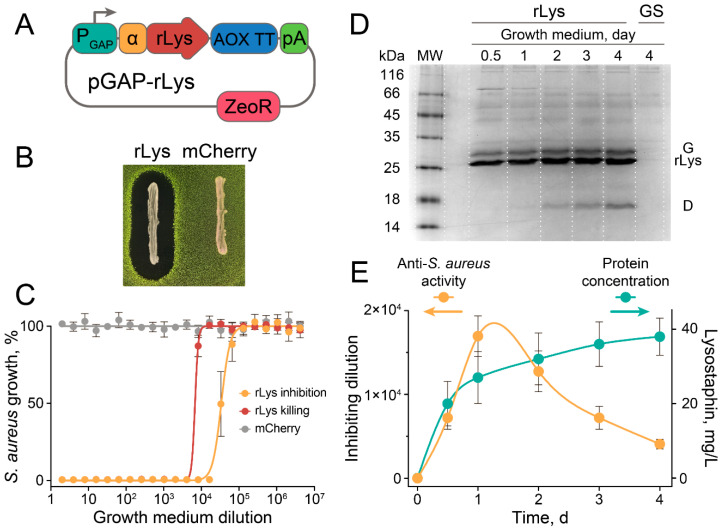
Production of recombinant lysostaphin (rLys) in *P. pastoris* and its anti-*S. aureus* activity. (**A**) Schematic representation of vector pGAPα-Lys engineered for heterologous constitutive production of rLys in *P. pastoris*. P_GAP_—glyceraldehyde-3-phosphate dehydrogenase (GAP) promoter, α—alpha-factor signal sequence, rLys—codon-optimized sequence of lysostaphin, AOX TT—AOX1 transcriptional terminator, pA—polyadenylation signal, ZeoR—zeocin resistance. (**B**) Agar overlay assay of *P. Pastoris* GS115 transformed with pGAPα-Lys (rLys) or control vector pGAPα-mCherry (mCherry). GFP-producing *S. aureus* was used as an indicator strain. (**C**) Inhibition of *S. aureus* growth by rLys *P. pastoris* culture medium. *S. aureus* growth was estimated by the fluorescence of GFP reporter. rLys inhibition (orange) and rLys killing (red) was measured after 1 day and 1 week, respectively. mCherry (gray) indicates the control medium obtained from mCherry-producing *P. Pastoris*. (**D**) rLys daily production into the growth medium, visualized by 15% PAGE. G—glycosylated rLys; D—a byproduct of rLys proteolytic degradation; GS—parent *P. pastoris* GS115 strain. rLys production was monitored in rLys *P. Pastoris* culture supernatant. (**E**) Anti-*S. aureus* activity in culture medium (orange) and protein production (aquamarine) time curves. Data represent the mean of three biological replicates ± SD.

The codon-optimized sequence of lysostaphin [25] was cloned into pGAPα under control of the strong constitutive glyceraldehyde-3-phosphate dehydrogenase (GAP) promoter, so as to maintain constitutive production of the recombinant lysostaphin in *P. pastoris* (Figure 1A). The *P. pastoris* transformed with pGAPα-Lys vector produced recombinant lysostaphin, and secreted it into the culture medium, giving clear zones of *S. aureus* inhibition in an agar overlay assay (Figure 1B).

The culture medium of rLys *P. pastoris* was highly active against *S. aureus*, leading to growth inhibition and bactericidal effects at 1.6 × 10^4^- and 4 × 10^3^-fold dilutions, respectively (Figure 1C). Control *P. pastoris* strain producing mCherry fluorescent protein did not influence *S. aureus* growth and viability (Figure 1B,C). *P. pastoris* produced up to 30 mg/L/day of recombinant lysostaphin in shaking flask conditions (Figure 1D,E), and we estimate that this productivity could be increased by several folds using bioreactor cultivation conditions. However, proteolysis of the recombinant lysostaphin was observed (Figure 1D), resulting in the maximal anti-*S. aureus* activity detected at the 24 h cultivation time point (Figure 1E). Since the specific activity of the rLys *P. pastoris* culture medium gradually decreased after 24 h of cultivation, we speculate that the production of recombinant lysostaphin by live rLys *P. pastoris* culture in situ is preferred for enhanced *S. aureus* eradication.

### 2.2. The Anti-Stapylococcal Activity of Engineered rLys P. pastoris

**Figure 2:**
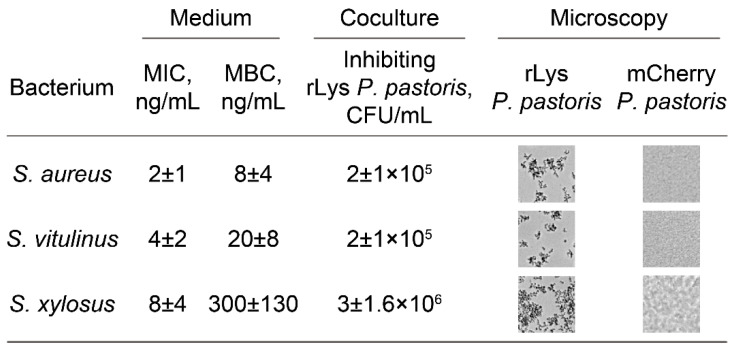
Antibacterial activity of rLys produced in a culture medium, and inhibitory activity of live rLys *P. pastoris* cell culture. The growth medium was collected after 1 d of cultivation. Live rLys *P. pastoris* cells were washed with a fresh medium before cocultivation with bacteria. ∼10^6^ CFU/mL of indicator bacteria was used. *Staphylococcus pasteuri*, *Staphylococcus warneri*, *Staphylococcus haemolyticus*, *Staphylococcus epidermidis*, *Macrococcus caseolyticus*, *Bacillus subtilis*, *Enterococcus faecium*, *Enterococcus faecalis* and *Escherichia coli* were not inhibited by rLys *P. pastoris* and rLys up to 50 µg/mL concentration. MIC—minimum inhibitory concentration, MBC—minimum bactericidal concentration.

Unlike a parent *P. pastoris* GS115 strain, engineered rLys *P. pastoris* displays antagonistic properties, mediated by constitutive lysostaphin production. To trace the correlation between lysostaphin activity spectrum and the antagonistic properties of rLys *P. pastoris* against different bacteria, we used a panel of *Staphylococci* and several other bacteria naturally resistant toward lysostaphin (Figure 2). The antagonistic properties of rLys *P. Pastoris* were similar to the lysostaphin activity spectrum. The most efficient growth inhibition was observed for *S. aureus* and *S. vitulinus*. Mediocre inhibition was observed for *S. xylosus.* Several *Staphylococci* (*Staphylococcus pasteuri*, *Staphylococcus warneri*, *Staphylococcus haemolyticus* and *Staphylococcus epidermidis*) and other bacteria (*Macrococcus caseolyticus*, *Bacillus subtilis*, *Enterococcus faecium*, *Enterococcus faecalis*, and *Escherichia coli*) were not inhibited by rLys *P. pastoris*. This is in line with the lysostaphin activity spectrum, and it is associated with the alternative PG structures among these bacteria [26].

Hence, engineered rLys *P. pastoris* displays quite a narrow activity spectrum. rLys *P. pastoris* acts efficiently on *S. aureus* and several *Staphylococci*, these having similar PG structures. This selectivity is favored for the practical application of rLys *P. pastoris* as it could be exploited as a targeted biocontrol agent for *S. aureus* eradication.

### 2.3. Eradication of S. aureus by Live rLys P. pastoris Biocontrol Agent

**Figure 3:**
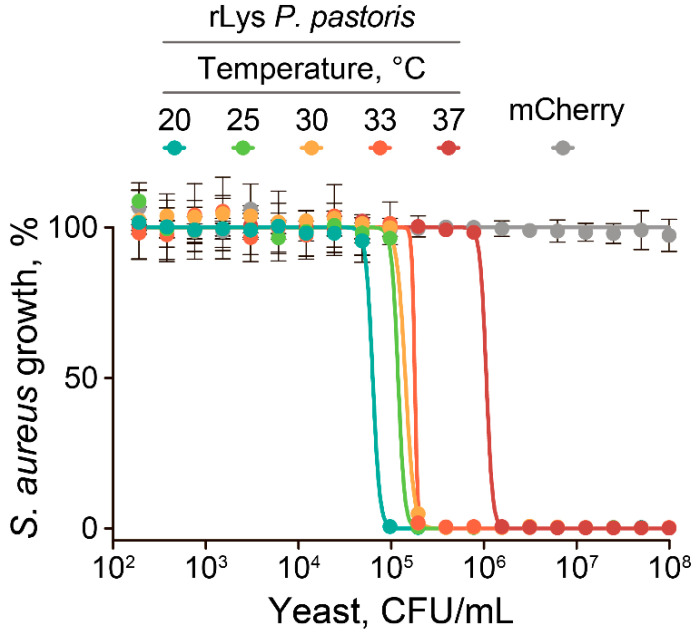
Growth temperature influences the inhibitory activity of the live rLys *P. pastoris* cell culture. The cocultivation of rLys *P. pastoris* with a GFP-producing *S. aureus* indicator strain was performed at different temperatures in the 20–37 °C range. *S. aureus* growth was estimated by the fluorescence of the GFP reporter. mCherry (gray) indicates control mCherry-producing *P. pastoris* strain. Data represent the mean of three biological replicates ± SD.

Focusing on the inhibitory properties of rLys *P. pastoris* cells alone, we screened different cocultivation modes of *S. aureus* and rLys *P. pastoris*. *S. aureus* and *P. pastoris* have different optimal growth temperatures, 37 °C and 30 °C, respectively. Hence, elevated temperatures could decrease the anti-*S. aureus* activity of rLys *P. pastoris* (Figure 3).

However, efficient *S. aureus* inhibition by rLys *P. pastoris* was observed up to 33 °C, corresponding to the temperature of the skin surface, which is an ecological niche of *S. aureus*. The further increase in cocultivation temperature resulted in an eight-fold decrease of anti-*S. aureus* activity at 37 °C (Figure 3). *S. aureus* inhibition was particularly efficient at temperatures below 25 °C, which is close to natural *P. pastoris* growth conditions.

**Figure 4:**
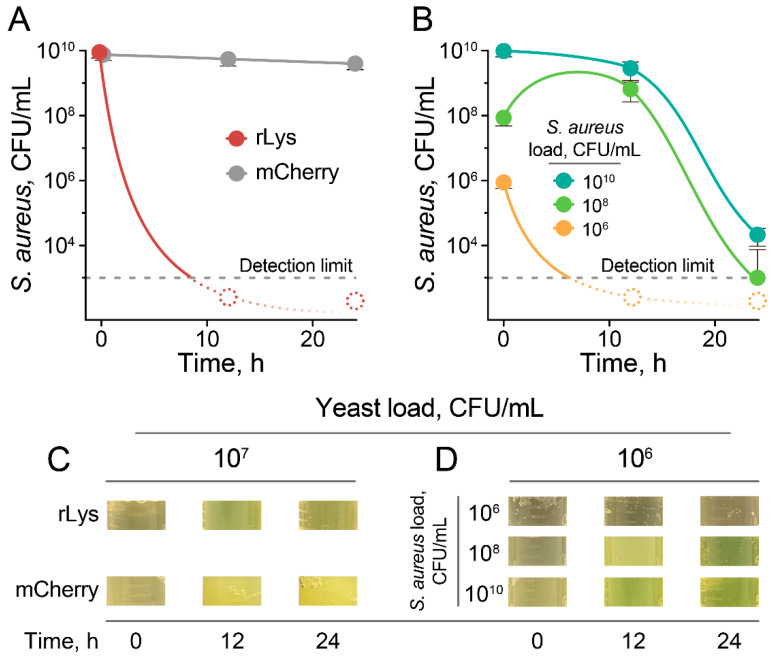
The cocultivation of *S. aureus* with rLys *P. pastoris* results in *S. aureus* killing. (**A**) Viability of *S. aureus* in coculture with 10^7^ CFU/mL of rLys *P. pastoris* (red) or control mCherry-producing *P. pastoris* (gray). Dashed dots indicate that no viable *S. aureus* was detected at the respective time point. The detection limit of viable *S. aureus* cells was ∼10^3^ CFU/mL. (**B**) Viability of *S. aureus* in coculture with 10^6^ CFU/mL of rLys *P. pastoris* at various *S. aureus* loads. 10^10^ (aquamarine), 10^8^ (green) and 10^6^ (orange) CFU/mL of *S. aureus* were used as a starting point. Data represent the mean of three biological replicates ± SD. (**C**,**D**) illustrate representative images of *S. aureus*/*P. pastoris* cocultures at various time points, corresponding to panels (**A**,**B**), respectively.

To estimate the kinetics of *S. aureus* eradication, we varied the target (*S. aureus*):effector (rLys *P. pastoris*) ratios and measured cell viability after cocultivation at 33 °C (Figure 4).

At relatively high rLys *P. pastoris* loads (10^7^ CFU/mL up to 10^10^ CFU/mL), *S. aureus* cells were killed within 12 h of cocultivation (Figure 4A). The control *P. pastoris* strain producing the mCherry fluorescent protein did not influence *S. aureus* viability, as was expected from the culture medium activity testing (Figure 4A). Decreasing the load of rLys *P. pastoris* to 10^6^ CFU/mL resulted in different scenarios. At a low *S. aureus* load, 10^6^ CFU/mL, rLys *P. pastoris* controlled *S. aureus* growth and eliminated viable cells within less than 12 h (Figure 4B). Increasing the *S. aureus* loads to 10^8^ CFU/mL resulted in a situation wherein rLys *P. pastoris* could not control *S. aureus* growth from the beginning of cocultivation. This led to *S. aureus* proliferation at the 12 h time point, while after that rLys *P. pastoris* produced enough lysostaphin to kill the majority of *S. aureus* cells, decreasing its titer by 5.8 orders of magnitude after 24 h of cocultivation (Figure 4B). In dense *S. aureus* cultures with loads up to 10^10^ CFU/mL, rLys *P. pastoris* gradually decreased *S. aureus* titer by 0.5 and 5.1 orders of magnitude at the 12 h and 24 h time points, respectively (Figure 4B). The decrease in viable *S. aureus* cells was associated with the massive *S. aureus* lysis detected by culture clearance (Figure 4C,D).

Thus, the complete or near-complete lysis of *S. aureus* cells is achieved by considerably low rLys *P. pastoris* concentrations at 12 h. *P. pastoris* cultures reach cell densities exceeding 10^10^ CFU/mL when cultivated in bioreactors. Hence, more than 1000-fold dilutions of rLys *P. pastoris* live cell cultures are efficient in *S. aureus* eradication. This makes it a safe and cost-effective biocontrol agent for eliminating *S. aureus* cells.

**Figure 5:**
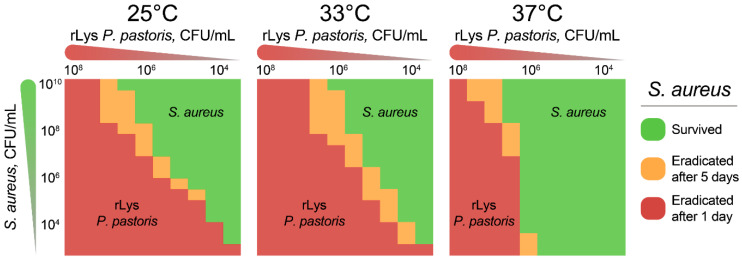
*S. aureus* eradication landscapes obtained by cocultivation with rLys *P. pastoris*. Various concentrations of *S. aureus* were cocultivated with rLys *P. pastoris*, resulting in *S. aureus* growth or eradication. Viable *S. aureus* cells were assayed after 1 day and 5 days. Areas indicating *S. aureus*/rLys *P. pastoris* ratios where live *S. aureus* was not detected after 1 day and 5 days are colored with red and orange, respectively. Areas with live *S. aureus* are colored with green. The detection limit of viable *S. aureus* cells is ∼10^3^ CFU/mL.

To further estimate the eradication landscapes of the rLys *P. pastoris* biocontrol agent, we performed time-lapse cocultivation of *S. aureus* and rLys *P. pastoris* at different target:effector ratios (Figure 5).

rLys *P. pastoris* eradicated *S. aureus* at various target:effector ratios at temperatures from 25 °C to 37 °C (Figure 5). However, the eradication of *S. aureus* was not observed at low (10^6^ CFU/mL) loads of rLys *P. pastoris* at 37 °C, indicating the necessity of effector proliferation for complete *S. aureus* removal (Figure 5). Both 25 °C and 33 °C temperatures were convenient for rLys *P. pastoris* growth resulting in *S. aureus* elimination even at yeast titers less than 10^4^ CFU/mL. However, only up to 10^4^ CFU/mL of *S. aureus* could be eradicated at such a low yeast level. The temperature of 25 °C resulted in a more efficient *S. aureus* eradication than 33 °C at low yeast loads, while this relation was the opposite at high effector concentrations (Figure 5). We associate this with the counteraction of the more efficient proliferation of rLys *P. pastoris* at low temperatures, and the increased bacteriolytic activity at high temperatures.

## 3. Discussion

*S. aureus* is a ubiquitous human bacterium with outstanding pathogenic potential. Infections that are caused by AR *S. aureus*, particularly MRSA strains, result in epidemic waves still presenting a global threat [4,5]. Conventional antibiotics are often inefficient in *S. aureus* infections nowadays. Potent probiotics antagonizing *S. aureus* could be an alternative, or could provide synergistic effects in *S. aureus* eradication. Efficient *S. aureus*-killing bacteria are presented in bacterial communities of human [27] and animal [28,29] origins. However, the native properties of these killing bacteria may considerably hinder their application as a probiotic and a safe biocontrol agent in vivo, or even make it dangerous [27]. Designer genetically programmed probiotics, on the other hand, are free of these side effects. Here we suggest engineering anti-*S. aureus* yeasts, since they are safe, simple to manipulate, and generally easily tolerate stress conditions. Similar to the majority of non-sporulating bacteria, *S. aureus* displays a rapid decrease in cell viability under starvation, resulting in 99–99.9% cell death after 2 days [30]. Unlike bacteria, yeasts have additional molecular pathways to adapt themselves to the various environmental conditions, e.g., introns promote yeast growth under starvation [31]. Hence, yeasts have an improved plasticity to survive under stress conditions and starvation, maintaining more than 50% survival for weeks and even months [32,33]. This is especially relevant to *P. pastoris*, which efficiently maintains viability by transcriptional reprogramming toward near-zero growth rates [34]. Thus, the genetically programmed *S. aureus*-killing yeast represents a proof-of-concept of the targeted designer yeast biocontrol agent.

rLys yeast efficiently eradicated *S. aureus* under a broad range of conditions and target:effector ratios. Even the 1000-fold numerical superiority of *S. aureus* over rLys *P. pastoris* in a dense culture resulted in *S. aureus* eradication. Moreover, complete *S. aureus* lysis could be achieved by cocultivation with only 10^7^ CFU/mL in less than 12 h. Trace amounts of rLys *P. pastoris* eliminated similar concentrations of *S. aureus* in coculture at 25–33 °C. The optimal growth temperature for *P. pastoris* is 28–30 °C. However, efficient *S. aureus* eradication was observed up to 33 °C. Even at 37 °C, the antagonistic effect of rLys *P. pastoris* was substantial. Although the reduction of *S. aureus*’ virulence factors at colder temperatures may take place [35] and influence eradication efficacy, we attribute the decreased efficiency of rLys *P. pastoris* at 37 °C to the impaired protein production, endoplasmic reticulum stress, and yeast cell death extensively reported previously [36,37]. Hence, rLys *P. pastoris* seems to be more suitable for *S. aureus* eradication from the environment or skin surfaces at temperatures around 33 °C. For in vivo applications based on oral probiotic administration, this methodology could be easily transferred to other non-pathogenic yeast strains displaying elevated growth temperatures. Several strains of *Saccharomyces* and *Candida* are culturable at temperatures up to 42 °C, providing this option, while strict safety precautions must be taken into account.

An extensive number of cryptic AR enzymes yet to be discovered [38] and resistance toward lysostaphin was documented for laboratory strains [19,39]. The major mechanism of acquired lysostaphin resistance relates to mutations in the *femA* gene, which encodes the factor responsible for the addition of the second and third glycines to the pentaglycine bridge of the cell wall [40]. However, lysostaphin-resistant variants of *S. aureus* demonstrate reduced fitness in vitro and in vivo [41]. Moreover, resistance to lysostaphin is associated with a loss of resistance toward β-lactams, resulting from a change in the PG crossbridge from pentaglycine to a single glycine [42]. Hence, the application of rLys yeast biocontrol agents may provide an artificial selective pressure for the amplification of *S. aureus* strains sensitive to conventional β-lactam antibiotics, thus passively targeting β-lactam AR. The combination of rLys yeast with β-lactam antibiotics may be another straightforward option, since lysostaphin and β-lactams have synergistic effects on oxacillin-resistant *Staphylococci* [43].

The efficient eradication of *S. aureus* by live rLys *P. pastoris* was achieved at high scales, resulting in a simple and cost-effective strategy for *S. aureus* lysis in bioproduction and surface decontamination. The reported strategy is easily scalable, providing an almost unlimited source of the biocompatible *S. aureus*-killing agent. Live rLys *P. pastoris* cultures diluted more than 1000 folds efficiently eradicate *S. aureus*. The exceptional viability of yeast, in turn, prevents subsequent re-colonization, serving as a selective artificial biocontrol agent. Future biomedical applications based on designer yeast biocontrol agents require their evaluation in vivo. However, we believe that this strategy is very promising, since it provides highly safe, efficient and selective genetically programmed probiotics and targeted biocontrol agents.

## 4. Materials and Methods

### 4.1. Bacterial and Yeast Strains

A bacterial collection of clinical isolates including *Enterococcus faecium*, *Enterococcus faecalis*, *Macrococcus caseolyticus*, *Staphylococcus vitulinus*, *Staphylococcus pasteuri*, *Staphylococcus warneri*, *Staphylococcus xylosus*, *Staphylococcus haemolyticus* and *Staphylococcus epidermidis* was kindly provided by Lytech Co. Ltd. (Moscow, Russia). *Escherichia coli* BL21(DE3), *Bacillus subtilis* 168 ATCC 23857 and clinical isolates were used as indicator bacteria in activity tests. Staphylococcus aureus, constitutively producing GFP, was described previously [27]. *Pichia pastoris* GS115 was used as a heterologous host for secreted lysostaphin production. rLys *P. pastoris* and the control mCherry-producing strain were obtained from *Pichia pastoris* GS115, transformed with pGAP-rLys or pGAP-mCherry, respectively.

### 4.2. Cultivation Conditions

*Pichia pastoris* was cultured in a 2YT medium (10 g/L yeast extract, 16 g/L tryptone, 5 g/L NaCl) at 30 °C in an orbital shaker at 200 rpm. All bacteria were grown in 2YT. The bacteria were inoculated with overnight culture using 1:100 dilutions, and were grown for 4 h at 37 °C and 250 rpm shaking. Chloramphenicol was added to a final concentration of 10 µg/mL for the *S. aureus* overnight culture.

### 4.3. Plasmid Construction and Yeast Transformation

Plasmid pGAPZalpha_BsmBI was constructed based on the pGAPZalpha A vector for the constitutive extracellular production of target proteins. An additional *BsmB*I restriction site was cloned into the MCS using annealed oligonucleotides. It enabled the insertion of a gene immediately downstream of the leader peptide. A codon-optimized lysostaphin sequence (GenBank: KF724949.1) was chemically synthesized and sub-cloned into the plasmid pGAPZalpha_BsmBI flanked by *BsmB*I/*Sal*I restriction sites. The resulting plasmid pGAP-rLys was linearized by the *Avr*II restriction site and the electroporated *P. pastoris* GS115 strain using lithium acetate and dithiothreitol [44]. The same procedure was done for a similar pGAP-mCherry construct. Transformed cells were selected on YPDS plates (2% peptone, 1% yeast extract, 2% glucose, 1M sorbitol, 2% agar) supplemented with 100 µg/mL of zeocin.

### 4.4. Recombinant Lysostaphin Production

The overnight culture of rLys *P. pastoris* was used to inoculate 100 mL 2YT to a final optical density at 600 nm (OD_600_) of 3, corresponding to 1.5 × 10^8^ CFU/mL. The yeast culture was grown for 96 h in 750 mL shake flasks. Aliquots were collected every 24 h, centrifuged at 5000 rpm for 10 min, and supernatants were stored at −80 °C. Protein concentration was estimated using comparative protein densitometry with a VersaDoc Molecular Imager MP 4000 system, followed by Quantity One software quantification and a Bradford Protein Assay of samples obtained by repetitive concentration with Amicon Ultra 10 kDa Centrifugal Filters.

### 4.5. Agar Overlay Assay

The agar overlay assay was conducted using soft-agar (5 g/L yeast extract, 8 g/L tryptone, 2.5 g/L NaCl, 0.7% agar, 10 µg/mL chloramphenicol). Tested *P. pastoris* strains were grown for 2 days on 2YT agar plates at 30 °C. Soft agar was melted, cooled to 42 °C, and inoculated with *S. aureus* using 1:2000 overnight culture dilution. A quantity of 5 mL of liquid soft agar was used to overlay *P. pastoris* streaks. Plates were incubated overnight at 30 °C after agar solidification.

### 4.6. Estimation of Lysostaphin Activity in Culture Media

The activity of culture media was tested in 96-well plates using serial dilutions in 2YT medium at 33 °C in a microplate shaker with 600 rpm shaking. The tested bacteria were diluted in 2YT medium to a final ~10^6^ CFU/mL. MICs were determined as the lowest concentration of lysostaphin that inhibited the growth of tested bacteria after 16 h of incubation. MBCs were determined as the lowest concentration of lysostaphin that inhibited the growth of the tested bacteria after 7 days of incubation. The bacterial growth time course was monitored by OD_600_ using a Varioskan Flash multimode reader. The growth of *S. aureus* was monitored using GFP fluorescence and OD_600_.

### 4.7. Cocultivation

The cocultivation analysis included the following: (i) rLys *P. pastoris* activity spectrum, (ii) temperature dependence of rLys *P. pastoris* activity, (iii) *S. aureus* killing kinetics, and (iv) *S. aureus* eradication landscapes. Both (i) and (ii) indicator bacterial strains were diluted in 2YT to ~10^6^ CFU/mL in 96-well plates. *P. pastoris* cells were washed with fresh 2YT and added to bacteria using various yeast concentrations in the 10^4^–10^8^ CFU/mL range. Plates were incubated in a microplate shaker at 33 °C and 600 rpm for 7 days. The bacterial growth was monitored using GFP fluorescence, OD_600_ and light microscopy. (ii) Plates were incubated at 20 °C, 25 °C, 30 °C, 33 °C and 37 °C for 7 days. (iii) *S. aureus*-killing kinetics were determined in 750 mL flasks at 33 °C with 200 rpm shaking. A quantity of 50 mL of 2YT was inoculated with microorganisms at different target:effector (*S. aureus*/rLys *P. pastoris*) titers: 10^10^/10^7^ CFU/mL, 10^10^/10^6^ CFU/mL, 10^8^/10^6^ CFU/mL and 10^6^/10^6^ CFU/mL. mCherry-producing *P. pastoris* was used in a 10^7^ CFU/mL concentration as a negative control. Aliquots were collected after 0, 12 and 24 h of incubation. Ten-fold serial dilutions were plated on 2YT agar plates supplemented with 10 µg/mL of chloramphenicol and incubated overnight at 37 °C for *S. aureus* count. (iv) Eradication landscapes were obtained by cocultivation of *S. aureus* and rLys *P. pastoris*, using three-fold serial dilutions starting from 10^10^ CFU/mL and 10^8^ CFU/mL, respectively. Plates were incubated at 25 °C, 33 °C and 37 °C for 5 days in a microplate shaker with 600 rpm shaking. The bacterial growth was monitored using GFP fluorescence, OD_600_ and light microscopy. The viability of *S. aureus* was assayed by plating, as was described above in (iii).

## 5. Conclusions

The constitutive heterologous production of highly specific bacteriolytic protease lysostaphin in yeast *Pichia pastoris* provides an efficient biocontrol agent, specifically killing *S. aureus* in coculture. Live rLys *P. pastoris* cultures diluted more than 1000 folds efficiently eradicate *S. aureus*. A yeast-based anti-*S. aureus* probiotic was efficient in a high range of temperatures and target-to-effector ratios, indicating its robustness and versatility in eliminating *S. aureus* cells. The efficient eradication of *S. aureus* by live rLys *P. pastoris* was achieved at high scales, resulting in a simple and cost-effective strategy for *S. aureus* lysis in bioproduction and surface decontamination. These results illustrate the considerable potential of live lysostaphin-producing cultures in *S. aureus* elimination, creating a background for their further applications in vivo.

## References

[1] Fleischmann C., Scherag A., Adhikari N.K.J., Hartog C.S., Tsaganos T., Schlattmann P., Angus D.C., Reinhart K. (2016). Assessment of Global Incidence and Mortality of Hospital-treated Sepsis. Current Estimates and Limitations. Am. J. Respir. Crit. Care Med..

[2] Rudd K.E., Johnson S.C., Agesa K.M., Shackelford K.A., Tsoi D., Kievlan D.R., Colombara D.V., Ikuta K.S., Kissoon N., Finfer S. (2020). Global, regional, and national sepsis incidence and mortality, 1990–2017: Analysis for the Global Burden of Disease Study. Lancet.

[3] Kourtis A.P., Hatfield K., Baggs J., Mu Y., See I., Epson E., Nadle J., Kainer M.A., Dumyati G., Petit S. (2019). Vital Signs: Epidemiology and Recent Trends in Methicillin-Resistant and in Methicillin-Susceptible *Staphylococcus aureus* Bloodstream Infections—United States. MMWR Morb. Mortal. Wkly. Rep..

[4] Chambers H.F., DeLeo F.R. (2009). Waves of resistance: *Staphylococcus aureus* in the antibiotic era. Nat. Rev. Microbiol..

[5] Turner N.A., Sharma-Kuinkel B.K., Maskarinec S.A., Eichenberger E.M., Shah P.P., Carugati M., Holland T.L., Fowler V.G. (2019). Methicillin-resistant *Staphylococcus aureus*: An overview of basic and clinical research. Nat. Rev. Microbiol..

[6] Harris S.R., Feil E.J., Holden M.T.G., Quail M.A., Nickerson E.K., Chantratita N., Gardete S., Tavares A., Day N., Lindsay J.A. (2010). Evolution of MRSA During Hospital Transmission and Intercontinental Spread. Science.

[7] Ochman H., Elwyn S., Moran N.A. (1999). Calibrating bacterial evolution. Proc. Natl. Acad. Sci. USA.

[8] Gibson B., Wilson D.J., Feil E., Eyre-Walker A. (2018). The distribution of bacterial doubling times in the wild. Proc. Biol. Sci..

[9] Waters E.M., Rowe S.E., O’Gara J.P., Conlon B.P. (2016). Convergence of *Staphylococcus aureus* Persister and Biofilm Research: Can Biofilms Be Defined as Communities of Adherent Persister Cells?. PLoS Pathog..

[10] Wu J.A., Kusuma C., Mond J.J., Kokai-Kun J.F. (2003). Lysostaphin disrupts *Staphylococcus aureus* and *Staphylococcus epidermidis* biofilms on artificial surfaces. Antimicrob. Agents Chemother..

[11] Schindler C.A., Schuhardt V.T. (1964). Lysostaphin: A New Bacteriolytic Agent for the *Staphylococcus*. Proc. Natl. Acad. Sci. USA.

[12] Gonzalez-Delgado L.S., Walters-Morgan H., Salamaga B., Robertson A.J., Hounslow A.M., Jagielska E., Sabała I., Williamson M.P., Lovering A.L., Mesnage S. (2020). Two-site recognition of *Staphylococcus aureus* peptidoglycan by lysostaphin SH3b. Nat. Chem. Biol..

[13] Bastos M.D., Coutinho B.G., Coelho M.L. (2010). Lysostaphin: A Staphylococcal Bacteriolysin with Potential Clinical Applications. Pharmaceuticals.

[14] Whitman W.B. (2015). Staphylococcus. Bergey’s Manual of Systematics of Archaea and Bacteria.

[15] Kusuma C.M., Kokai-Kun J.F. (2005). Comparison of four methods for determining lysostaphin susceptibility of various strains of *Staphylococcus aureus*. Antimicrob. Agents Chemother..

[16] Yang X.Y., Li C.R., Lou R.H., Wang Y.M., Zhang W.X., Chen H.Z., Huang Q.S., Han Y.X., Jiang J.D., You X.F. (2007). In vitro activity of recombinant lysostaphin against *Staphylococcus aureus* isolates from hospitals in Beijing, China. J. Med. Microbiol..

[17] Placencia F.X., Kong L., Weisman L.E. (2009). Treatment of methicillin-resistant *Staphylococcus aureus* in neonatal mice: Lysostaphin versus vancomycin. Pediatr. Res..

[18] Dajcs J.J., Hume E.B., Moreau J.M., Caballero A.R., Cannon B.M., O’Callaghan R.J. (2000). Lysostaphin treatment of methicillin-resistant *Staphylococcus aureus* keratitis in the rabbit. Investig. Ophthalmol. Vis. Sci..

[19] Climo M.W., Patron R.L., Goldstein B.P., Archer G.L. (1998). Lysostaphin treatment of experimental methicillin-resistant *Staphylococcus aureus* aortic valve endocarditis. Antimicrob. Agents Chemother..

[20] Kokai-Kun J.F., Chanturiya T., Mond J.J. (2007). Lysostaphin as a treatment for systemic *Staphylococcus aureus* infection in a mouse model. J. Antimicrob. Chemother..

[21] Johnson C.T., Wroe J.A., Agarwal R., Martin K.E., Guldberg R.E., Donlan R.M., Westblade L.F., García A.J. (2018). Hydrogel delivery of lysostaphin eliminates orthopedic implant infection by *Staphylococcus aureus* and supports fracture healing. Proc. Natl. Acad. Sci. USA.

[22] Kerr D.E., Plaut K., Bramley A.J., Williamson C.M., Lax A.J., Moore K., Wells K.D., Wall R.J. (2001). Lysostaphin expression in mammary glands confers protection against staphylococcal infection in transgenic mice. Nat. Biotechnol..

[23] Liu Y., Bai P., Woischnig A.K., Charpin-El Hamri G., Ye H., Folcher M., Xie M., Khanna N., Fussenegger M. (2018). Immunomimetic Designer Cells Protect Mice from MRSA Infection. Cell.

[24] Blazanovic K., Zhao H., Choi Y., Li W., Salvat R.S., Osipovitch D.C., Fields J., Moise L., Berwin B.L., Fiering S.N. (2015). Structure-based redesign of lysostaphin yields potent antistaphylococcal enzymes that evade immune cell surveillance. Mol. Ther. Methods Clin. Dev..

[25] Zhao H., Blazanovic K., Choi Y., Bailey-Kellogg C., Griswold K.E. (2014). Gene and protein sequence optimization for high-level production of fully active and aglycosylated lysostaphin in *Pichia pastoris*. Appl. Environ. Microbiol..

[26] Vollmer W., Blanot D., De Pedro M.A. (2008). Peptidoglycan structure and architecture. FEMS Microbiol. Rev..

[27] Terekhov S.S., Smirnov I.V., Stepanova A.V., Bobik T.V., Mokrushina Y.A., Ponomarenko N.A., Belogurov A.A., Rubtsova M.P., Kartseva O.V., Gomzikova M.O. (2017). Microfluidic droplet platform for ultrahigh-throughput single-cell screening of biodiversity. Proc. Natl. Acad. Sci. USA.

[28] Terekhov S.S., Smirnov I.V., Malakhova M.V., Samoilov A.E., Manolov A.I., Nazarov A.S., Danilov D.V., Dubiley S.A., Osterman I.A., Rubtsova M.P. (2018). Ultrahigh-throughput functional profiling of microbiota communities. Proc. Natl. Acad. Sci. USA.

[29] Terekhov S.S., Nazarov A.S., Mokrushina Y.A., Baranova M.N., Potapova N.A., Malakhova M.V., Ilina E.N., Smirnov I.V., Gabibov A.G. (2020). Deep Functional Profiling Facilitates the Evaluation of the Antibacterial Potential of the Antibiotic Amicoumacin. Antibiotics.

[30] Watson S.P., Clements M.O., Foster S.J. (1998). Characterization of the starvation-survival response of *Staphylococcus aureus*. J. Bacteriol..

[31] Parenteau J., Maignon L., Berthoumieux M., Catala M., Gagnon V., Abou Elela S. (2019). Introns are mediators of cell response to starvation. Nature.

[32] Petti A.A., Crutchfield C.A., Rabinowitz J.D., Botstein D. (2011). Survival of starving yeast is correlated with oxidative stress response and nonrespiratory mitochondrial function. Proc. Natl. Acad. Sci. USA.

[33] Sideri T., Rallis C., Bitton D.A., Lages B.M., Suo F., Rodríguez-López M., Du L.-L., Bähler J. (2014). Parallel profiling of fission yeast deletion mutants for proliferation and for lifespan during long-term quiescence. G3.

[34] Rebnegger C., Vos T., Graf A.B., Valli M., Pronk J.T., Daran-Lapujade P., Mattanovich D. (2016). *Pichia pastoris* Exhibits High Viability and a Low Maintenance Energy Requirement at Near-Zero Specific Growth Rates. Appl. Environ. Microbiol..

[35] Anderson K.L., Roberts C., Disz T., Vonstein V., Hwang K., Overbeek R., Olson P.D., Projan S.J., Dunman P.M. (2006). Characterization of the *Staphylococcus aureus* Heat Shock, Cold Shock, Stringent, and SOS Responses and Their Effects on Log-Phase mRNA Turnover. J. Bacteriol..

[36] Li Z., Xiong F., Lin Q., d’Anjou M., Daugulis A.J., Yang D.S.C., Hew C.L. (2001). Low-Temperature Increases the Yield of Biologically Active Herring Antifreeze Protein in *Pichia pastoris*. Protein Expr. Purif..

[37] Zhong Y., Yang L., Guo Y., Fang F., Wang D., Li R., Jiang M., Kang W., Ma J., Sun J. (2014). High-temperature cultivation of recombinant *Pichia pastoris* increases endoplasmic reticulum stress and decreases production of human interleukin-10. Microbial. Cell Factories.

[38] Terekhov S.S., Mokrushina Y.A., Nazarov A.S., Zlobin A., Zalevsky A., Bourenkov G., Golovin A., Belogurov A., Osterman I.A., Kulikova A.A. (2020). A kinase bioscavenger provides antibiotic resistance by extremely tight substrate binding. Sci. Adv..

[39] Strandén A.M., Ehlert K., Labischinski H., Berger-Bächi B. (1997). Cell wall monoglycine cross-bridges and methicillin hypersusceptibility in a femAB null mutant of methicillin-resistant *Staphylococcus aureus*. J. Bacteriol..

[40] Ehlert K., Schröder W., Labischinski H. (1997). Specificities of FemA and FemB for different glycine residues: FemB cannot substitute for FemA in staphylococcal peptidoglycan pentaglycine side chain formation. J. Bacteriol..

[41] Kusuma C., Jadanova A., Chanturiya T., Kokai-Kun J.F. (2007). Lysostaphin-resistant variants of *Staphylococcus aureus* demonstrate reduced fitness in vitro and in vivo. Antimicrob. Agents Chemother..

[42] Climo M.W., Ehlert K., Archer G.L. (2001). Mechanism and suppression of lysostaphin resistance in oxacillin-resistant *Staphylococcus aureus*. Antimicrob. Agents Chemother..

[43] Kiri N., Archer G., Climo M.W. (2002). Combinations of lysostaphin with beta-lactams are synergistic against oxacillin-resistant *Staphylococcus epidermidis*. Antimicrob. Agents Chemother..

[44] Wu S., Letchworth G.J. (2004). High efficiency transformation by electroporation of *Pichia pastoris* pretreated with lithium acetate and dithiothreitol. BioTechniques.

